# Supplementary material for: Repeated Transcranial Photobiomodulation with Light-Emitting Diodes Improves Psychomotor Vigilance and EEG Networks of the Human Brain
Source: Bioengineering (Basel). 2023 Sep 5;10(9):1043. doi: 10.3390/bioengineering10091043 (PMC10525861; doi:10.3390/bioengineering10091043)
Supplement: Supplementary file 1 [file bioengineering-10-01043-s001.zip › bioengineering-2525425-supplementary.pdf]

bioengineering

MDPI

Supplementary Materials

Repeated Transcranial Photobiomodulation with Light-Emitting Diodes Improves Psychomotor Vigilance and EEG Networks of the Human Brain

Akhil Chaudhari, Xinlong Wang, Anqi Wu and Hanli Liu *

Department of Bioengineering, University of Texas at Arlington, 500 UTA Blvd, Arlington, TX 76019, USA; akhilmchaudhari@gmail.com (A.C.); xl.victor.wang@gmail.com (X.W.); anqi.wu@uta.edu (A.W.)
* Correspondence: hanli@uta.edu

A. Identification of human EEG networks and their alterations by longitudinal tPBM

A newly developed algorithm has been published that enables the identification of human EEG networks, as represented in 2-dimentional (2D) topographies and 3-dimensional (3D) cortical source locations for each network [36]. The essence of this algorithm is the combination of group singular value decomposition (gSVD) with exact low-resolution brain electromagnetic tomography (eLORETA). The overall processing steps are shown in Figure 3 in the main text of the paper. More details for each processing step is provided below.

Step 1: Pre-processing of raw EEG data

We followed the same procedures described in the sub-section of "*EEG data preprocessing*" of the main paper.

Steps 2: Z-score transformation

Group SVD can be considered similar to spatial group independent component analysis (ICA), which has been widely used in the field of fMRI [50-52]. The pre-processed EEG data were initially standardized by performing z-score transformations to minimize inter-subject variations. Specifically, we had each preprocessed EEG time series in a selected time period subtract its own temporal mean and then divided it by its temporal standard deviation. This standardization step was necessary for an unbiased operation in gSVD.

Step 3: Forming the group for gSVD calculations

The standardized EEG time series from all 32 samples of the EEG measurements (n = 7 and 9 participants for the tPBM and sham groups in S1 of week 1; n = 8 and 8 for both groups in S8 of week 4, respectively) were concatenated into a single 2D matrix, $\mathbf{M}_{gSVD}$. One dimension of this 2D matrix was the concatenated time covering TP1 and TP2 in both S1 and S8 for the sham and LED groups. The other dimension was 64 channels with standardized EEG readings. There are interconnections between slow and fast EEG rhythms in mediating the brain networks [53]. Hence, the five frequency bands of EEG (delta, theta, alpha, beta, and gamma) were considered together, rather than separating them into individual frequency bands, while performing gSVD.

Steps 4 and 5: Computations of gSVD across EEG measures in S1 and S8 from both groups

gSVD was performed on the concatenated matrix, $\mathbf{M}_{gSVD}$, to identify the common Principal Components (PCs) across the four weeks and two groups with the native MATLAB function 'svd'. Specifically, gSVD was performed using the 'economy-size decomposition' style to remove extra rows and columns of zeros in the time dynamics vector

(U) and singular-value vector (S). The mathematical equation of the 'svd' function can be expressed as in Eq. 1:

$$C = U \times S \times V^T \quad (1)$$

where C is the transposed matrix of $\mathbf{M}_{gSVD}$ (i.e., C = $\mathbf{M}_{gSVD}^T$); S is a diagonal matrix containing 64 singular values of C with a decreasing magnitude, thus marking the weight of each component in C; U is an orthogonal matrix and represents the time dynamics vectors for all 64 singular components without a unit; and V is an orthogonal matrix with a dimension of 64 × 64 (64 channels of 64 singular components). Matrix V facilitated 2D topographies for all 64 singular components without a unit (owing to the z-score transformation of the EEG data). As a result, we obtained 64 gSVD-derived PCs with their respective weights (from matrix S) over the original signal, their corresponding 1D time series for each of the key components (from matrix U), and the corresponding topography for each respective component (from matrix V). Consequently, we selected all components that had less than a 90% reduction in the most-weighted components, as well as extracted 12 gSVD components to form a 2D topography and for the 3D source localization analysis. These 12 components were used to define the 12 EEG brain networks, which may change their electrophysiological powers through the longitudinal LED-tPBM during PVT performance.

Step 6: Source localization using eLORETA

The 3D cortical source localizations were projected using eLORETA for the 12 identified gSVD components. eLORETA is a free-access software package (http://www.uzh.ch/keyinst/loreta.htm) that converts the 2D scalp distribution of the electrical potential into a 3D distribution of the current density in the human brain. To localize the electrical activity in the human cortex, eLORETA uses a total of 6239 voxels at a 5 mm spatial resolution. eLORETA offers a weighted least-squares based solution with a localization error [54]. In this study, eLORETA was utilized to localize the 3D cortical sources (cortical space) of the 2D electric potential distribution (sensor space) of the 12 SVD components. The Montreal Neurological Institute (MNI) coordinate system of the 64-electrode international 10-10 system was employed, and a default value of 1 was utilized as a regularization parameter for the generation of the transformation matrix. This procedure produced 3D cortical maps; in addition, 2D (sagittal, coronal, and axial) views were also generated for each SVD component.

To specifically examine the power changes in the EEG brain networks induced by longitudinal LED-tPBM during PVT performance, we divided the 1D time series (matrix U) into separate temporal segments for TP1 and TP2 and for S1 of Week 1 and S8 of Week 4 in the data process. This process was repeated for each participant and each group (LED and Sham). Accordingly, the power spectral density calculations for all the 12 components/networks were performed on the segmented EEG brain networks for each participant, as described in Step 7.

Step 7: Calculations of the PSD of the 12 SVD components

Quantification of the PSDs for each of the 12 SVD components (i.e., brain EEG networks) during TP1 and TP2 in S1 of Week 1 and S2 of Week 4 for both groups was achieved based on respective the time courses obtained using the native MATLAB function "pwelch" (with a 20 sec window and 50% overlap). This operation resulted in one PSD curve with a resolution of 0.125 Hz, ranging from 0 to 128 Hz for each SVD component, each subject, each week, and for each TP (TP1 and TP2).

Step 8: Computation of the power changes of EEG networks induced by 4-week LED-tPBM

To compute the absolute power change of the 12 EEG networks induced by the 4-week LED-tPBM stimulation, we obtained the spectrally averaged SPD power by

multiplying the averaged PSD value over the corresponding spectral band; this was conducted with the respective frequency bandwidth for each subject for each of the 5-*f* bands (Delta: 0.5-4 Hz, Theta: 4-7 Hz, Alpha: 7-13 Hz, Beta: 13-30 Hz, and Gamma: 30-70 Hz) during TP1 and TP2 in Sessions S1 and S8. Next, each frequency-specific power at each of the 5-*f* bands was baseline-normalized with respect to its own frequency-specific baseline power for all 12 SVD components, for all subjects in both the tPBM and sham groups, and for TP1 and TP2 in both S1 and S8. The group-averaged nPSD values were obtained in each sham and tPBM group for all 12 components or networks. Furthermore, we computed the differences in the normalized network powers between S8 in Week 4 and S1 in Week 1 (i.e., Δnp = np_S8 – np_S1) for each of the sham and tPBM groups in all 5-f bands during TP1 and TP2, respectively. Finally, significant differences in the ΔnP between the tPBM versus sham groups were determined by performing two-sample, non-parametric tests [55,56] during TP1 and TP2 for each component in each frequency band at the significance level of $p < 0.05$ (marked by "*") and $p < 0.01$ (marked by "&"). A MATLAB function of "ranksum" was used to perform the non-parametric permutation comparisons between the LED and sham groups in the 5 frequency bands and the 12 brain EEG networks.

B. Topographies of the longitudinal effects induced by 4-week LED-tPBM

The longitudinal effects were obtained by taking the differences between sham-subtracted nPSD (ss-nPSD) topographies in Week 1 and the respective ones in Week 4 for all 5-*f* bands during TP1 and TP2.As shown in Figure S1, during TP1, a 60% increase in ss-nPSD was observed in the left and right temporal regions in the gamma band after 4 weeks of LED stimulation with respect to those in Week 1. During TP2, an 80% increase in ss-nPSD was observed globally in the theta, beta, and gamma bands, while the delta band showed a significant increase in the right temporal and medial lobes. An 80% increase was also observed in right temporal and left occipital regions for alpha band.

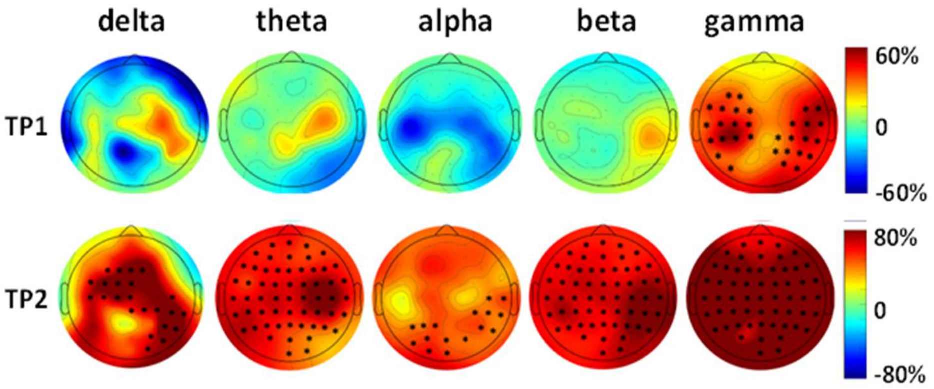

Figure S1. Topographic maps of the ss-nPSD changes in EEG power after 4-week tPBM during TP1 and TP2. The columns represent the delta, theta, alpha, beta, and gamma frequency bands, respectively. The first and second rows correspond to the 4-week, tPBM-induced ss-nPSD changes during TP1 and TP2 in Session 8, respectively. Color bars indicate the percent changes in ss-nPSD with respect to those in Session 1 of Week 1. The dark symbols within each topographic map mark the corrected p value < 0.05 after performing the cluster-based permutation test for multiple comparisons.
